# El mandato transformador del Sistema Interamericano como respuesta a la pandemia a la luz del test democrático The Inter-American System’s transformative mandate as a response to the pandemic in light of the democratic test

**DOI:** 10.1093/icon/moab111

**Published:** 2022-02-07

**Authors:** Mariela Morales Antoniazzi

**Keywords:** collective guarantee, inter-American democratic test, interamericanization, *ius constitutionale commune*

## Abstract

This article analyzes how Latin American democracies should react to the COVID-19 pandemic. From the perspective of a ius constitutionale commune in Latin America, it argues that the greatest challenge lies in passing the inter-American democratic test, which at the intersection of human rights, democracy and the rule of law can guarantee compliance with inter-American standards. A new rationale derives from the mechanisms adopted by the Inter-American Court of Human Rights and the Inter-American Commission on Human Rights, in the exercise of the transformative mandate in times of pandemic. It concludes by identifying a “double” democratic test in Latin America that relies on the notion of collective guarantee of human rights standards.

## Abstract (es)

El presente artículo analiza cómo deben reaccionar las democracias latinoamericanas frente a la pandemia del COVID-19. Desde la perspectiva de un ius constitutionale commune en América Latina, se argumenta que el mayor desafío radica en aprobar el test democrático interamericano, que en la intersección de los derechos humanos, la democracia y el estado de derecho pueda garantizar el cumplimiento de los estándares interamericanos. Un nuevo fundamento deriva de los mecanismos adoptados por la Corte Interamericana de Derechos Humanos y la Comisión Interamericana de Derechos Humanos, en ejercicio del mandato transformador en tiempos de la pandemia. Se concluye identificando un doble test democrático en América Latina que se apoya en la noción de garantía colectiva de los estándares de derechos humanos.

En el contexto de la pandemia causada por el COVID-19, América Latina ha reflejado sus múltiples crisis y evidenciado, una vez más, las desigualdades, la discriminación y la exclusión de personas y grupos en situación de vulnerabilidad. Asimismo, las respuestas estatales dirigidas a enfrentar la pandemia han implicado riesgos para la democracia y el estado de derecho.^1^ Sin embargo, el argumento que presento se basa en afirmar que en la esfera jurídico-política es clave aprobar el “test democrático interamericano” en la región, no solo para avanzar hacia una sociedad más resiliente y resistente en el respeto de los estándares democráticos en los estados de excepción,^2^ sino también para abordar una relectura de los principios de indivisibilidad e interdependencia de los derechos humanos.^3^ Los mecanismos innovadores de la Comisión Interamericana de Derechos Humanos^4^ confirman el potencial del impacto transformador del Sistema Interamericano de Derechos Humanos (SIDH). Al definir las líneas rojas para los estados parte de la Convención Americana (CADH)^5^ y, además, al defender la solidaridad regional y global, los mecanismos se convierten en una referencia fundamental para la protección de la democracia bajo los estándares del Derecho internacional.

## 1. La protección de la democracia como desafío multinivel

Existen tres desafíos centrales en la región de América Latina: Primero, la adopción de medidas excepcionales para hacer frente a la pandemia supuso, en la mayoría de los casos, la restricción de derechos y la concentración de poderes adicionales en el ejecutivo.^6^ En las democracias constitucionales, en el Derecho internacional y en el derecho interamericano, las declaratorias de estados de excepción deben cumplir los principios de legalidad, proporcionalidad, necesidad y temporalidad, y respetar el acervo común construido en el *case law*. Igualmente, la separación de poderes, sus controles y sus equilibrios adquieren aún mayor relevancia al considerar la larga tradición de autoritarismo, hiper-presidencialismo y debilidad de la institucionalidad, aunado más recientemente a las protestas sociales y las crisis políticas.^7^ En consecuencia, el reto es que la actuación a nivel estatal salvaguarde la tríada democracia, estado de derecho y derechos humanos y facilite buenas prácticas en la interamericanización. Segundo, representa un desafío comprender la defensa de la democracia y la garantía de los derechos humanos desde su doble condicionalidad y enlazada con la protección multinivel. En el espacio jurídico latinoamericano, se trata de resistir la pandemia cumpliendo el test democrático interamericano para garantizar que las medidas adoptadas en la lucha contra el COVID-19 se ajusten a los estándares de protección de los derechos humanos, en el marco de una sociedad democrática, por mandato constitucional y convencional. Tercero, la pandemia lleva consigo dependencias globales y la necesidad de un enfoque transversal y holístico en materia de derechos humanos. Los estados, en función de sus obligaciones para garantizar la igualdad en el disfrute del derecho a la salud y el derecho a la vida, los Derechos Económicos, Sociales, Culturales y Ambientales (DESCA) y los derechos civiles y políticos, igualmente están anclados en la búsqueda de la equidad en las estrategias de vacunación, debido al desafío en la intersección entre las desigualdades a nivel nacional y las enormes disparidades en los ámbitos regional y mundial. Es esencial que el Derecho internacional desempeñe un papel protagónico en el acceso universal y equitativo a las vacunas contra el COVID-19, a fin de evitar que las vacunas oscilen entre la “proeza científica y el fracaso moral”.^8^

## 2. El test democrático como marco analítico

El *corpus iuris democrático* en la región de América Latina se expresa básicamente en la Declaración Americana^9^ y la Convención Americana, así como en la Carta Democrática Interamericana (CDI).^10^ Este instrumento, que cumple dos décadas en este 2021, emergió como una herramienta para actualizar, interpretar y aplicar la Carta de la OEA^11^ en materia democrática e implica un desarrollo progresivo del Derecho internacional. Se estableció una concordancia de los contenidos normativos en aras de ofrecer el concepto común de la democracia, como conjunto de valores mínimos de su régimen jurídico en el SIDH. Desde ese *corpus iuris* se deduce lo que denomino el test democrático interamericano y se articula a partir de los elementos esenciales y componentes fundamentales de la consolidación democrática (arts. 3 y 4), de una condicionalidad dual entre democracia y derechos humanos (arts. 7 y 8), en interdependencia con lo social (arts. 11 a 13). Una reconstrucción normativa del sistema interamericano permite comprobar un reforzamiento evolutivo de la concepción dual y social de la democracia, que da luces para guiar la actuación de los estados en tanto constituye una premisa indispensable para el ejercicio efectivo de las libertades fundamentales y los derechos humanos, en su carácter universal, indivisible e interdependiente, consagrados en el *ius commune* (constituciones de los estados e instrumentos interamericanos e internacionales de derechos humanos), todo bajo la reafirmación de los estados miembros de fortalecer el sistema interamericano de protección de los derechos humanos para la consolidación de la democracia en el Hemisferio. Por lo tanto, defender la democracia mediante el test interamericano es una responsabilidad constitucional y convencional.

El test democrático ejemplifica el potencial del acervo de un *ius constitutionale commune* en América Latina (ICCAL),^12^ para promover una relación de refuerzo mutuo entre los estados y los órganos del SIDH, ratificar la coadyuvancia entre ambos niveles y habilitar a los diversos actores en torno al conjunto de normas comunes, para propiciar transformaciones estructurales.^13^ El impacto transformador de la CIDH y la Corte IDH se revela según la forma en que se entiende y aplica el Derecho internacional y los respetivos instrumentos de derechos humanos a nivel regional. No cabe duda de que, los órganos del SIDH han reiterado sus estándares a fin de que los estados parte de la CADH, no solo aprueben el test democrático, sino favorezcan el proceso de interamericanización de los órdenes nacionales, enraizados en los valores, principios y estándares universales en el contexto de la pandemia.

## 3. Las respuestas de la Corte IDH y de la CIDH – consolidando el mandato transformador

La Declaración 1/20 emitida por la Corte IDH, en cuanto al COVID-19 y los Derechos Humanos, insta a los estados a adoptar e implementar medidas que estén dentro del marco del estado de derecho, con pleno respeto de los instrumentos interamericanos para la protección de los derechos humanos y los estándares desarrollados en su jurisprudencia. La Corte IDH insiste en que resulta indispensable la garantía del “acceso a la justicia y a los mecanismos de denuncia, así como se proteja particularmente la actividad de las y los periodistas y las defensoras y defensores de derechos humanos”, todo ello conforme a su jurisprudencia para preservar los estándares de una sociedad democrática. La Corte IDH advierte también que, en razón de la naturaleza de la pandemia, los DESCA “deben ser garantizados sin discriminación a toda persona bajo la jurisdicción del Estado y, en especial, a aquellos grupos que son afectados de forma desproporcionada porque se encuentran en situación de mayor vulnerabilidad”. Con este Declaración, de manera pionera e innovadora, se reafirma la vigencia del test democrático interamericano, interpretando conjuntamente el *corpus iuris* con las exigencias de la Carta Democrática, condición *sine qua non* para el abordaje de la pandemia.

Del mismo modo, la CIDH ha ejercido su mandato transformador frente a la pandemia, particularmente con las resoluciones 1/20 “Pandemia y Derechos Humanos en las Américas”, la Resolución 4/20 “Derechos Humanos de las personas con COVID-19” y 1/21 “Las vacunas contra el COVID-19 en el marco de las obligaciones interamericanas de derechos humanos”. Ambos instrumentos, junto con la SACROI COVID-19, los comunicados de prensa y el monitoreo en la región, revelan el enfoque holístico de los derechos humanos. La Resolución 1/2020, con sus 85 recomendaciones destinadas a los estados, enfatiza el deber de los estados de adoptar un enfoque centrado en los derechos humanos en toda estrategia, política o medida estatal. A más de un año de la crisis sanitaria y sus múltiples efectos en todas las esferas de la vida, la Resolución 1/2021 atiende la urgencia de garantizar una rápida inmunización y aclara el deber de los estados de proteger la salud pública en aras del acceso equitativo a las vacunas, concebidas como un bien público universal y regional. Este importante reconocimiento incentiva la revisión de las desigualdades estructurales que la pandemia exacerbó y que el *ius commune* del test democrático ofrece las pistas y rutas para enfrentarlo.

## 4. Perspectivas del mandato transformador: el doble test democrático

El test democrático interamericano, en los términos previstos en la Carta Democrática en interpretación conjunta con el acervo del *ius commune* construido en la región, se ha fortalecido con las innovaciones del SIDH en el ejercicio de su mandato transformador bajo la perspectiva de entender la defensa de la democracia como un reto que solo se puede asumir desde una protección multinivel de los derechos humanos.

Tanto en la Resolución 01/20 de la CIDH como en la Declaración 01/20 de la Corte IDH se subrayó que los estados tienen la obligación de respetar y garantizar las normas internacionales, de proteger las libertades fundamentales y el estado de derecho, así como de garantizar la independencia de los órganos y mecanismos de control del estado, al igual que los DESCA y los derechos civiles y políticos, con un deber de protección reforzada para las personas y grupos en situación de vulnerabilidad. Un paso más se alcanza con la Resolución 01/21 de la CIDH al incluir la novedosa perspectiva de la solidaridad como factor imprescindible para la adecuada superación de la pandemia. Todo ello implica para los Estados la adecuación de la normativa interna mediante la propia interamericanización derivada del test democrático interamericano.

Una tercera perspectiva en clave de los estándares de la sociedad democrática en su vínculo intrínseco con los derechos humanos se deriva de lo que puede denominarse el doble test democrático, con fundamento en los mecanismos de garantía colectiva, como salvaguardas esenciales adscritas a la configuración de un estado democrático, puestos de relieve por la Corte IDH en su Opinión Consultiva N° 26 cuando se pronunció respecto a la denuncia de la CADH y de la Carta de la OEA.^14^ Estos mecanismos de garantía colectiva deben activarse contra denuncias intempestivas y contrarias al principio general del derecho de actuar de buena fe. La Corte IDH, al pronunciarse acerca el escrutinio sobre la buena fe estatal en conexión con el propósito y contexto en que se gesta y verifica la denuncia, enuncia supuestos “que denotan una especial gravedad y pueden acarrear una afectación a la estabilidad democrática, la seguridad y la paz hemisférica, con la consiguiente afectación generalizada a los derechos humanos”. Es un nuevo test democrático expresado en la garantía colectiva, en convergencia con el mandato transformador del SIDH. Sin duda, la Opinión Consultiva N° 28^15^ reitera la necesaria vigencia de la interdependencia entre democracia, estado de derecho y derechos humanos para guiar las respuestas de los estados particularmente en estos tiempos de pandemia.

## Footnotes

1 Carlos Ayala Corao, *Retos de la pandemia del Covid-19 para el estado de derecho, la democracia y los derechos humanos* (*The Challenges that the Pandemic Caused by Covid-19 Has Presented for the Rule of Law, Democracy, and Human Rights*) (Max Planck Institute for Comparative Public Law & International Law MPIL, Research Paper No. 2020-17, 1, 2020).

2 Armin von Bogdandy, Jesús María Casal & Mariela Morales Antoniazzi, *La resistencia del Estado democrático de Derecho en América Latina frente a la pandemia de COVID-19. Un enfoque desde el ius commune* 76-87 (Max Planck Institute for Comparative Public Law & International Law MPIL, Research Paper No. 2020-35, 2020):

3 Mariela Morales Antoniazzi & Flavia Piovesan, *Interdependencia e indivisibilidad de los derechos humanos: Una nueva mirada frente al covid-19*, Anuario de Derechos Humanos. 35 (2020).

4 COVID-19 y Derechos Humanos: los problemas y desafíos deben ser abordados con perspectiva de derechos humanos y respetando las obligaciones internacionales, Declaración 1/20, Corte IDH (9 de abril de 2020); Pandemia y Derechos Humanos en las Américas, Resolución 1/20, CIDH (10 de abril de 2020); Derechos Humanos de las Personas con COVID-19, Resolución 4/20, CIDH (27 de julio de 2020); Las vacunas contra el COVID-19 en el marco de las obligaciones interamericanas de derechos humanos, Resolución 1/21, CIDH (6 de abril de 2021).

5 Convención Americana sobre Derechos Humanos, adoptada el 22 de noviembre de 1969, entró en vigencia el 18 de julio de 1978.

6 Roberto Gargarella & Jorge Roa Roa, *Diálogo democrático y emergencia en América Latina* (*Democratic Dialogue and Emergency in Latin America*) 11-15 (Max Planck Institute for Comparative Public Law & International Law MPIL, Research Paper No. 2020-21, 2020).

7 Merike Blofield, Bert Hoffmann, & Mariana Llanos, *Assessing the Political and Social Impact of the COVID-19 Crisis in Latin America* (German Institute for Global and Area Studies, GIGA Focus Lateinamerika, Research Paper No. 3, 2020).

8 Rodrigo Uprimny Yepes, *Vacunas: proeza científica y fracaso moral*, Dejusticia (18 de abril de 2020), https://www.dejusticia.org/column/vacunas-proeza-cientifica-y-fracaso-moral/ (último acceso: 7 de septiembre de 2021).

9 Declaración Americana de los Derechos y Deberes del Hombre, adoptada en abril de 1948, entró en vigencia el 2 de mayo de 1948.

10 Carta Democrática Interamericana, adoptada el 11 de septiembre de 2001, entró en vigencia el 11 de septiembre de 2001.

11 Carta de la Organización de los Estados Americanos, adoptada el 30 de abril de 1948, entró en vigencia el 13 de diciembre de 1951.

12 Armin von Bogdandy, *Ius Constitutionale Commune en América Latina. Aclaración conceptual*, en Ius Constitutionale Commune en América Latina. Textos básicos para su comprensión 137 (Armin von Bogdandy, Mariela Morales Antoniazzi & Eduardo Ferrer Mac-Gregor coords., 2017).

13 1 Test democrático interamericano frente al COVID-19 (Mariela Morales Antoniazzi ed., 2021).

14 La denuncia de la Convención Americana sobre Derechos Humanos y de la Carta de la Organización de los Estados Americanos y sus efectos sobre las obligaciones estatales en materia de derechos humanos, Opinión Consultiva OC-26/20, Corte IDH (ser. A) No. 26 (9 de noviembre de 2020).

15 La figura de la reelección presidencial indefinida en sistemas presidenciales en el contexto del sistema interamericano de derechos humanos, Opinión Consultiva OC-28/21, Corte IDH (ser. A) No. 28 (7 de junio de 2021).

